# Supplementary material for: Evaluation of Clinical and Biochemical Traits in Egyptian Barki Sheep with Different Growth Performances
Source: Animals (Basel). 2023 Mar 7;13(6):962. doi: 10.3390/ani13060962 (PMC10044517; doi:10.3390/ani13060962)
Supplement: Supplementary file 1 [file animals-13-00962-s001.zip › animals-2252265-supplementary.pdf]

Supplemental information file

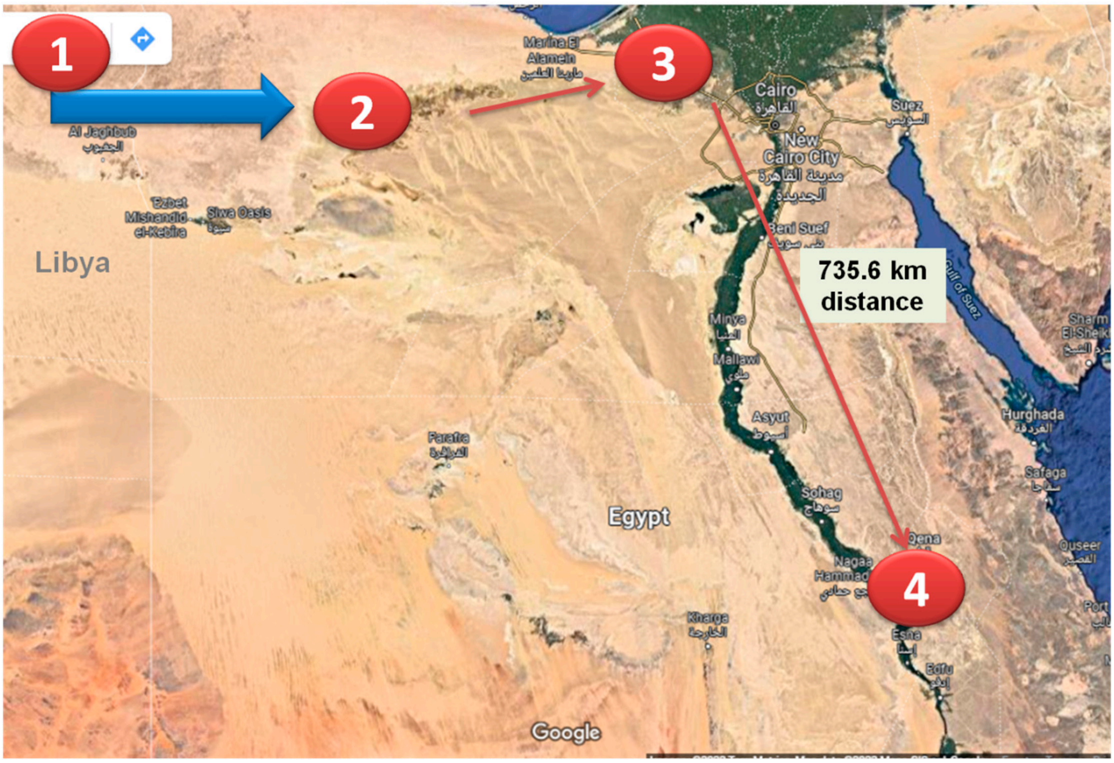

Figure S1. Map of Egypt showing the location and movement of Barki sheep of this study. 1; refers to Barca region in Eastern Libya (origin of Barki sheep in Egypt), 2; Matrouh governorate which is the most common place for breeding of Barki sheep in Egypt, 3; Beheira governorate which is the main collection center for Egyptian Barki sheep and the main distributing region for other Egyptian governorates, 4; Qena governorate which was the final destination for our tested Barki sheep.

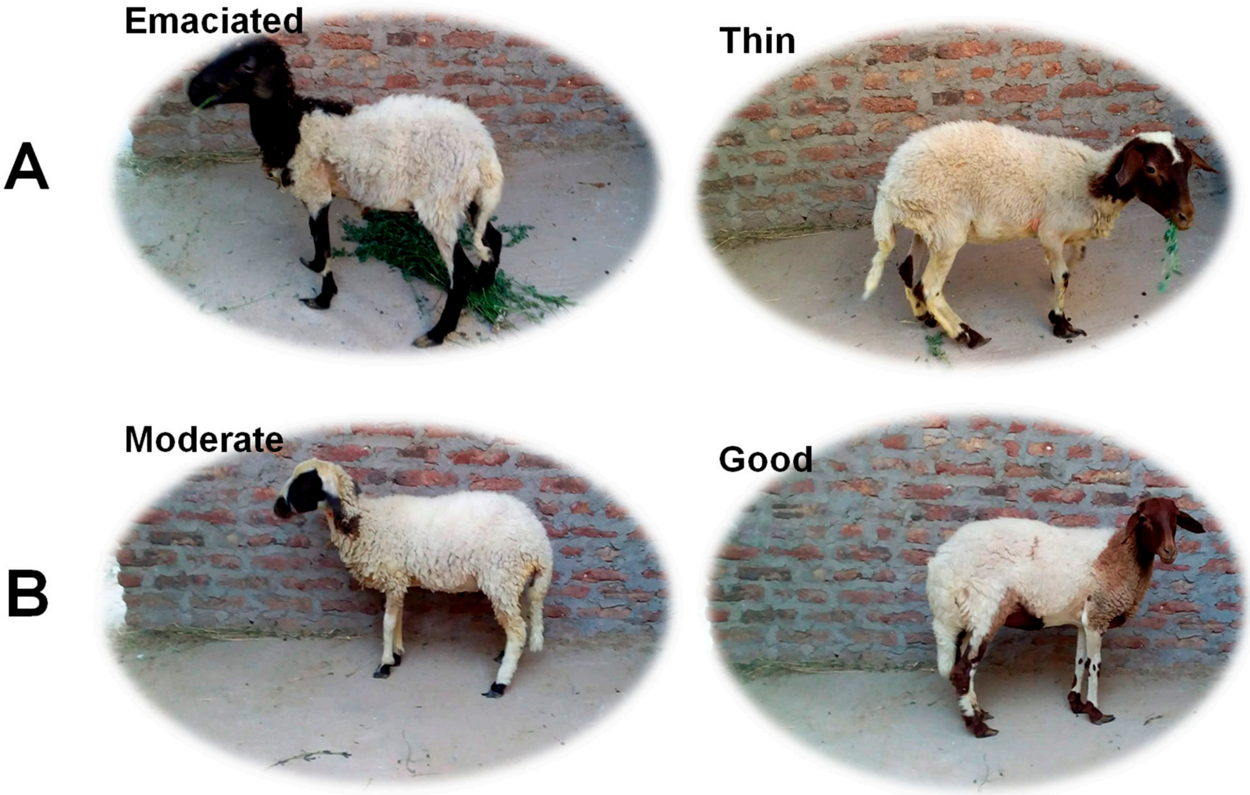

Figure S2. Representative image for Barki sheep used in the current study. (A) Sheep of stunted or test group that included animals with body condition score of BCS 1; emaciated and BCS 2; thin. (B) Sheep of good body condition or control group that comprised animals with body condition score of BCS 3; moderate and BCS 4; good.

Table S1. Biochemical variables among stunted and good body condition Barki sheep.

Parameter (unit)	Stunted (n = 10)	Good condition (n = 9)	*P- value*
Cholesterol (mg/dl)	35.7 ±5.8	49.8 ±8.8	0.0007
Triglycerides (mg/dl)	36 ±3.3	46.9 ±10.4	0.0059
Creatinine (mg/dl)	0.58 ±0.09	0.81 ±0.1	<0.0001
Urea (mg/dl)	29.6 ±3.2	26.9 ±2.9	0.0381
Blood urea nitrogen (mg/dl)	13.8 ±1.5	12.3 ±1.4	0.0381
Total bilirubin (mg/dl)	0.5 ±0.29	0.46 ±0.16	0.738
Direct bilirubin (mg/dl)	0.15 ±0.07	0.13 ±0.03	0.473
Indirect bilirubin (mg/dl)	0.35 ±0.3	0.33 ±0.15	0.858
ALT (U/l)	31.1 ±5.4	35.1 ±12.6	0.370
AST(U/l)	86.5 ±46.4	90.8 ±33	0.822
Total protein (g/dl)	6.7 ±0.44	7.2 ±0.48	0.0445
Albumin (g/dl)	3.6 ±0.27	4.1 ±0.4	0.0087
Globulin (g/dl)	3.1 ±0.42	3.1 ±0.7	1
A/G ratio (numerical)	1.2 ±0.2	1.4 ±0.4	0.193
Glucose (mg/dl)	45.3 ±5.3	45.1 ±9.1	0.956
Calcium (mg/dl)	8.7 ±0.6	8.3 ±2.4	0.700
Phosphorus (mg/dl)	5.7 ±0.6	5.9 ±0.9	0.432
Magnesium (mg/dl)	2.2 ±0.2	2.4 ±0.2	0.096
Iron (μg/dl)	111.8 ±25	127.6 ±16.2	0.124
Zinc (μg/dl)	50.3 ±25.3	44 ±11.2	0.499
TSH (IU/ml)	0.003 ±0.002	0.005 ±0.005	0.342
Growth hormone (ng/ml)	0.06 ±0.03	0.08 ±0.04	0.250

ALT; Alanine transaminase, AST; Aspartate transaminase, TSH; Thyroid-stimulating hormone. Significant values at p <0.05 and highly significant at p <0.0001 as measured by t-test (unpaired)
